# Roles and Mechanisms of Irisin in Attenuating Pathological Features of Osteoarthritis

**DOI:** 10.3389/fcell.2021.703670

**Published:** 2021-09-28

**Authors:** Xiangfen Li, Xiaofang Zhu, Hongle Wu, Thomas E. Van Dyke, Xiaoyang Xu, Elise F. Morgan, Wenyu Fu, Chuanju Liu, Qisheng Tu, Dingming Huang, Jake Chen

**Affiliations:** ^1^State Key Laboratory of Oral Diseases and National Clinical Research Center for Oral Diseases, West China Hospital of Stomatology, Sichuan University, Chengdu, China; ^2^Division of Oral Biology, Tufts University School of Dental Medicine, Boston, MA, United States; ^3^Clinical and Translational Research, Forsyth Institute, Cambridge, MA, United States; ^4^Oral Medicine, Infection, and Immunity, Harvard School of Dental Medicine, Boston, MA, United States; ^5^Department of Chemical and Materials Engineering, New Jersey Institute of Technology, Newark, NJ, United States; ^6^Department of Mechanical Engineering, Boston University, Boston, MA, United States; ^7^Department of Orthopedics Surgery, New York University School of Medicine, New York, NY, United States; ^8^Department of Cell Biology, New York University School of Medicine, New York, NY, United States

**Keywords:** irisin, osteoarthritis, cartilage, gene knockout, transgenics

## Abstract

To investigate the effects and mechanisms of irisin, a newly discovered myokine, in cartilage development, osteoarthritis (OA) pathophysiology and its therapeutic potential for treating OA we applied the following five strategical analyses using (1) murine joint tissues at different developmental stages; (2) human normal and OA pathological tissue samples; (3) experimental OA mouse model; (4) irisin gene knockout (KO) and knock in (KI) mouse lines and their cartilage cells; (5) *in vitro* mechanistic experiments. We found that Irisin was involved in all stages of cartilage development. Both human and mouse OA tissues showed a decreased expression of irisin. Intra-articular injection of irisin attenuated ACLT-induced OA progression. Irisin knockout mice developed severe OA while irisin overexpression in both irisin KI mice and intraarticular injection of irisin protein attenuated OA progression. Irisin inhibited inflammation and promoted anabolism in chondrogenic ADTC5 cells. Proliferative potential of primary chondrocytes from KI mice was found to be enhanced, while KO mice showed an inhibition under normal or inflammatory conditions. The primary chondrocytes from irisin KI mice showed reduced expression of inflammatory factors and the chondrocytes isolated from KO mice showed an opposite pattern. In conclusion, it is the first time to show that irisin is involved in cartilage development and OA pathogenesis. Irisin has the potential to ameliorate OA progression by decreasing cartilage degradation and inhibiting inflammation, which could lead to the development of a novel therapeutic target for treating bone and cartilage disorders including osteoarthritis.

## Introduction

Osteoarthritis (OA) is a chronic joint disease characterized by the degeneration of cartilage and subchondral bone within the joint. It afflicts about 10% of the population (Lawrence et al., 2008) and is currently a major cause of pain and disability worldwide. The major pathological changes of OA present in three ways: cartilage degradation and degeneration, aberrant subchondral bone metabolism, and synovial inflammation. However, the underlying mechanisms for the development of these changes are not fully understood.

Currently, there is no complete cure available for OA, only treatments designed to temporarily relieve pain and improve function. Conservative treatments, including weight loss, physical therapy/exercise, and activity modification, may not always elicit desired outcomes due to three major therapeutic limitations of conservative treatments: unsatisfactory clinical efficacy for pain relief, the potential for side effects with certain drug options, and the inability to delay disease progression (Zhang et al., 2011a). Interventional treatments include intra-articular (IA) injections and surgery. IA therapies seem likely to be more effective than systemic pharmacologic treatments such as non-steroidal anti-inflammatory drugs (NSAIDs), but part of the observed benefit of IA might be placebo effects (Yan et al., 2011). Surgical options include arthroscopic debridement, lavage and meniscectomy, high tibial osteotomy, and uni-compartmental and total knee arthroplasty (TKA). In joint replacement surgery (arthroplasty), surgical risks include infections and blood clots. Artificial joints can wear out or come loose and may eventually need to be replaced (Zhang et al., 2011a). Many of the current therapeutics for OA have limitations, drawbacks and side effects. Therefore, investigating the pathogenesis of OA and searching for an effective and robust therapy to prevent and treat OA is important and significant.

Irisin, a newly discovered myokine, is a polypeptide hormone derived from the proteolytic cleavage of fibronectin-type III domain-containing 5 (FNDC5) protein (Boström et al., 2012; Zhang et al., 2017). Previous studies have demonstrated that irisin has beneficial effects in various tissues and organs particularly in bone development and regeneration (Colaianni et al., 2015; Qiao et al., 2016; Mazur-Bialy et al., 2017a; Eslampour et al., 2019; Natalicchio et al., 2019). Irisin injections can improve cortical bone mineral density and skeletal remodeling (Colaianni et al., 2015, 2017). Also, irisin may suppress the pro-inflammatory activation of adipocyte 3T3 L1 cells and has potential anti-inflammatory properties connected with the downregulation of downstream pathways of TLR4/MyD88 (Mazur-Bialy et al., 2017b). Generally, there are both abnormal subchondral bone structures and enhanced inflammation in the OA joints. Here, we established the surgically induced OA mouse model and irisin genetic and transgenic mouse lines to investigate the roles of irisin in subchondral bone deterioration prevention, cartilage erosion containment and inflammation inhibition. Meanwhile, we have also initially validated its therapeutic potential for treating and reversing the OA pathologic features.

## Materials and Methods

### Animals

We have generated floxed irisin conditional knockout mice (IRS^f/f^) (Zhu et al., 2021) and irisin over-expressing mice R26^IRS/IRS^, which were both on the C57BL/6J background. To created R26^IRS/IRS^ mice, we constructed a transgenic cassette that carries a floxed stop codon fused to mouse IRS cDNA with a polyadenylation signal, under the control of the CAG promoter. The cassette was inserted into the intron1 of the Rosa26 locus. The function of the chimeric gene (floxed Stop-FNDC5-2A-tdTomato) was validated before microinjection. Transgene microinjection was performed at Biocytogen LLC (Worcester, MA) into C57BL/6 fertilized mouse eggs. We obtained C57BL/6J wide type mice and CMV-Cre mice from the Jackson Laboratory (stock No: 006054). To generate transgenic CMV^Cre+^/R26^IRS/IRS^ mice and CMV^Cre+^/R26^–/–^, R26^IRS/IRS^ mice were mated with CMV^Cre+^ mice to obtain CMV^Cre+^/R26^IRS/–^ mice, which were then interbred. To generate CMV^Cre+^/IRS^f/f^ mice and CMV^Cre+^/IRS^–/–^ mice, IRS^f/f^ mice were mated with CMV^Cre+^ mice to obtain CMV^Cre+^/IRS^f/–^ mice, which were then interbred. Animals were housed in a condition at 23 ± 2°C, with 50 ± 10% humidity and a 12 h light-12 h dark cycle (light on from 07:00 to 19:00) with the number of 5 mice per cage. Mice can get free access to regular rodent chow and water while be monitored three times a week for health status at the Tufts Medical Center Animal Facility. At the start of the experiments, mice weighed (mean ± SD) 22 ± 2 g. The study was based on previous experiments performed by other researchers (the same model but different knockout genes) to estimate an effect size on Osteoarthritis Research Society International (OARSI) score of 2.1 (Yu et al., 2020). To test the genes expression change in OA patient cartilages compared with the normal human, the results of other genes of the same sample types tested in our laboratory suggested the effect size of 2.6. Then we chose three normal human articular cartilage samples and 6 OA patients to test the mRNA expression level of irisin. This study was approved by the Institutional Animal Care and Use Committee (IACUC) of Tufts University in accordance with NIH guidelines.

### Cell Culture

Murine articular cartilages were isolated from CMV^Cre+^/R26^IRS/IRS^, CMV^Cre+^/R26^–/–^, CMV^Cre+^/IRS^f/f^, and CMV^Cre+^/IRS^–/–^ mice (*n* = 6), respectively, and the mice were 2 days old. Primary murine chondrocytes were cultured as previously described (Gosset et al., 2008). Briefly, we placed the mice in the face-down position and fixed the anterior legs by needles. We removed the skin and soft tissues using scissors and princer. Then, dislocated the femurs and collected the femoral heads, femoral condyles and tibial plateau. Cartilages were separated and incubated in 10 ml digestion solution (3 mg/ml collagenase D) for 45 min at 37°C. Then, retrieved the cartilages and repeated the digestion step. After that, we retrieved the cartilages and put them into 0.5 mg/ml collagenase D overnight at 37°C. Finally, collected the suspension of isolated cells and cells were seeded at 3 × 10^5^/well in 6-well culture plates in DMEM supplemented (Thermo Fisher Scientific Inc., Waltham, MA) with 10% fetal bovine serum (Thermo Fisher Scientific Inc.), 2% L-GIn (Sigma-Aldrich, St. Louis, MO) and 1%PS (penicillin and streptomycin, Thermo Fisher Scientific Inc.). ADTC5 cells (ATCC, Manassas, VA) were seeded at 1.2 × 10^5^/well in 12-well culture plates and 3 × 10^5^/well in 6-well culture plates in growth medium [Nutrient Mixture F-12, F-12/DMEM (1:1, Thermo Fisher Scientific Inc.) + 5% FBS + 1%PS + 10 μg/mL human transferrin (Sigma-Aldrich) + 30 nM sodium selenite (Sigma-Aldrich)]. To induce chondrogenic differentiation of ADTC5, we changed the growth medium to differentiation medium [Growth medium + 10 μg/mL insulin (Sigma-Aldrich) + 37.5 μg/mL ascorbic acid (Sigma-Aldrich)]. The cell culture medium was added into 10 ng/mL human recombinant proteins interleukin-1 beta (rhIL-1β, R&D Systems, Minneapolis, MN) to induce inflammation. Recombinant irisin (r-IRS, 100 ng/mL, AdipoGen, San Diego, CA) was added to the medium to detect its function *in vitro*, using the same volume PBS as control.

### Human Normal Cartilage and OA Tissue Samples

Normal human articular cartilage was isolated from the knees of patients (*n* = 3) who had died of diseases unrelated to arthritis (specimens obtained *en bloc* from the Musculoskeletal Transplant Foundation). The Kellgren-Lawrence Grading System was used to determine the grade of osteoarthritis (Kellgren and Lawrence, 1957). Normal cartilage samples were without radiographic or intra-articular evidence of arthritic disease (Kellgren-Lawrence Grade 0). Arthritic cartilages were obtained from patients (*n* = 6) undergoing elective total knee arthroplasty for end-stage OA with Kellgren-Lawrence Grade of 3 or 4 from the distal femora of 8 patients (mean age 58.4 years, range 49–66 years). The samples were then stored at −80°C until analysis (Luan et al., 2008). In this study the relevant samples and cDNA extracted from human cartilage were obtained from Dr. Chuanju Liu’s Lab at the NYU Medical School with an IRB protocol number of 12758.

### Mouse Limb Tissues at Different Developmental Stages

The limbs were isolated from embryonic (E14.5 and E16.5), new-born (D1, D4 and D7), adolescent C57BL/6J wide type mice (D28) (*n* = 6). Subsequently, the limbs were fixed in 4% buffered paraformaldehyde for 24 h, decalcified in 10% EDTA, then embedded in paraffin.

### OA Mouse Models and Intraarticular Injection of r-IRS

Twelve-week-old adult male C57BL/6J wide type mice (*n* = 12) were randomly divided into two groups [anterior cruciate ligament transection (ACLT)-operated treated with PBS, and ACLT-operated treated with irisin] and anesthetized through intraperitoneal injection of both xylazine (5 mg/kg, Akorn, Lake Forest, IL) and ketamine (40 mg/kg, Zoetis, Parsippany, NJ). Then OA was surgically induced by ACLT on the right knee, leaving the left knee as a sham surgery control in which the meniscotibial ligament is localized but not transected as previously described (Kamekura et al., 2005; Glasson et al., 2007; Zhao et al., 2014). At 2 days post-OA surgery, intraarticular injection into the knee joint was performed by a trans-patellar tendon approach. Five microliters of r-IRS (1 μg/μl) or PBS were injected every 3 days in the laboratory. Mice were returned to the cage until the day of next injection. The experimental mice were sacrificed at 8 weeks after the surgery. Surgically induced destabilization of the medial meniscus (DMM) model was performed on the transgenic mice. Twelve-weeks-old adult male transgenic mice from the groups (CMV^Cre+^, CMV^Cre+^/R26^IRS/IRS^, CMV^Cre+^/IRS^f/f^, CMV^Cre+^/IRS^f/f^ + IRS; *n* = 10 in each group) were anesthetized and unilateral joint instability will be induced by microsurgical transection of the medial meniscotibial ligament (MMTL) as previously described (Zhao et al., 2015). The DMM surgery were completed on the right knee. The polyester-based hydrogel was synthesized following previously published procedures (Tsou et al., 2018) with minor modifications. Briefly, the CHPO-Ser-ET oligomer was synthesized via polycondensation reaction between citric acid, serine and hexaethylene glycol. Polyester oligomers and 8-arm PEG-Maleimide (MW 10 KDa) were dissolved individually in PBS with r-IRS (1 μg/μl, pH = 7.4, AG-40B-0103, AdipoGen, San Diego, CA) to form pre-gel solutions with predetermined weight concentrations (5, 10%). By mixing two solutions, hydrogels were formed at physiological pH and temperature. The hydrogels with r-IRS were sent to evaluate the drug release profiles from hydrogel. Briefly, the IRS-loaded hydrogel was immersed in PBS. The suspension liquid was collected and analyzed for the release study using ELISA following the methods established in our lab and others that are designed specifically for IRS analysis (Efe et al., 2017; Tsou et al., 2018; Yuan et al., 2019). At 2 days post-OA surgery, the hydrogel (10 wt%, 1XPBS, pH = 7.4, 1 μg/μl IRS) was injected into the knee joints of KO mice by a trans-patellar tendon approach every 3-days in the laboratory. The experimental mice were sacrificed at 8 weeks after the surgery. The researchers participating in this study were blinded to the r-IRS treatment while analyzing and processing the data.

### Assessment of Progression and Severity of Osteoarthritis

After being decalcified with 10% EDTA for 2 weeks, the mouse knee joint samples were processed into paraffin-embedded sections. To determine the extent of cartilage deterioration, the human cartilage and mouse knee joint sections were examined by hematoxylin and eosin (H&E) staining and Safranin-O staining (StatLab, McKinney, TX) as we previously described (Valverde et al., 2008). Images were obtained by a Nikon Eclipse 300 fluorescence microscope (Compix Media Inc., Irvine, CA). A semi-quantitative histological scoring system issued by OARSI was used for evaluating the OA severity as previously described (Glasson et al., 2010). Three sections from each mouse were randomly selected for quantification. The OARSI scoring was performed in a blinded manner by two researchers in our lab. The inter-rater reliability was indicated by the ICC (intraclass correlation coefficient). In our experiments, the ICC was 0.942 ± 0.015, which indicated that the scoring system used in our experiments is reliable. The OARSI scoring results in each group were presented in form of mean ± SD, which suggested the inter-experimental error in the following comparison of differences between groups.

### Immunohistochemistry

To detect if irisin is involved in cartilage development, we collected the cartilage and surrounding tissues from embryonic, new-born, adolescent and adult mice, and performed immunohistochemistry (IHC) studies using irisin antibody (AG-25B-0027-C100, AdipoGen, San Diego, CA). The knee joints of transgenic mice were collected and performed IHC studies using COL2a1 (collagen type II alpha 1 chain) antibody (ab34712, Abcam, Cambridge, MA), Aggrecan antibody (ab186414, Abcam, Cambridge, MA) and COLX (collagen type X) antibody (14-977-82, Thermo Fisher Scientific, Waltham, MA). Images were obtained by a Nikon Eclipse 300 fluorescence microscope (Compix Media Inc.). Immunostaining positive chondrocytes in each field were counted. Three sections from each mouse were randomly selected for quantification.

### Alcian Blue Staining

To induce chondrogenic differentiation, the ADTC5 cells were incubated in chondrogenic differentiation medium for 2 weeks. The cells were fixed with 4% polyoxymethylene for 15 min, then stained with 1% Alcian blue (Sigma Aldrich).

### Cell-Counting Kit (CCK8) Assay

The ADTC5 cells were seeded in 96-well plates at a density of 3,000 cells/well in triplicate for each time point and unseeded wells were used as background controls. To determine the cell viability, a 5-day time course was implemented, and a 10-μl CCK-8 solution was added daily and incubated for 2 h. The absorbance at 450 nm was measured by a microplate reader as we previously described (Li et al., 2020).

### RNA Extraction and Quantitative Real-Time Polymerase Chain Reaction (qPCR)

Total RNA was extracted by TriZol (Invitrogen, Carlsbad, CA). cDNA was generated using oligo(dT)20 primer (Life Technologies, Carlsbad, CA) and SuperScript III reverse transcriptase (Life Technologies). Then, RNA quantification was performed using SYBR Green Supermix (Bio-Rad, Hercules, CA) on the Bio-Rad iQ5 thermal cycler according to the manufacturer’s protocol. The fold increase in PCR products by 2^–ΔΔ^Ct method was calculated using the housekeeping gene GAPDH as we previously described (Li et al., 2020).

### Statistical Analysis

The data of the experiments are presented as means ± SD. The significance of differences in various categorical variables was evaluated using one-way analysis of variance (ANOVA) for multi-group comparisons and the *t-*test for two-group comparison. For all quantitative assays, each assay condition was repeated in triplicate. A value of *P* < 0.05 was considered statistically significant.

## Results

### Irisin Is Involved in the Chondrogenesis and Shows Decreased Expression in Both Human and Mouse OA

**FIGURE 1:**
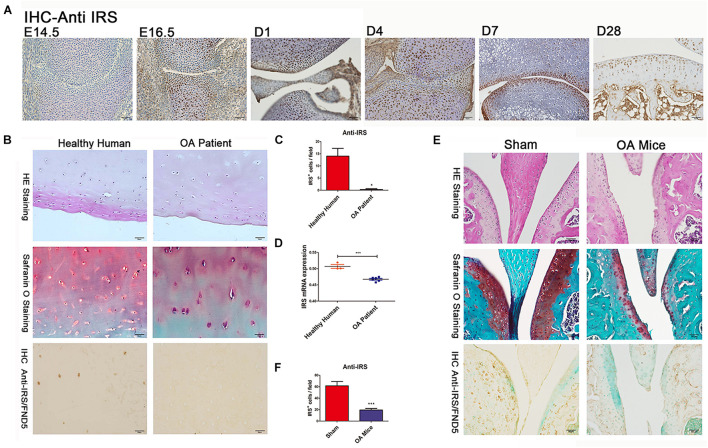
Irisin is involved in chondrogenesis and osteoarthritis (OA). **(A)** IHC shows irisin expression in cartilage cells at different developmental stages with distinct differential patterns (C57BL/6J wide type mice, *n* = 6). **(B,C)** The articular cartilage of OA patients (*n* = 6) showed a decreased expression of irisin (hematoxylin and eosin staining, Safranin-O staining, and immunohistochemical analyses). **(D)** Cartilage of OA patients (*n* = 6) exhibits decreased irisin mRNA expression compared with healthy humans (*n* = 3). **(E,F)** The articular cartilage of OA mice showed a decreased expression of irisin (C57BL/6J wide type mice with ACLT-model, *n* = 6). *p < 0.05, ***p < 0.001.

We collected the cartilages and surrounding tissues from embryonic, new-born, adolescent mice, and performed immunohistochemical studies. We found for the first time that irisin is prominently expressed in the prehypertrophic and hypertrophic zones of growth plate cartilage as early as E14.5 and E16.5 (Figure 1A), while expression was absent in the proliferating and resting zones at those stages. The expression of irisin was also present in the articular cartilage of postnatal mice (Figure 1A). Irisin was expressed throughout all zones of the cartilage at D1 and D4. Later at D7 and particularly D28, irisin was mainly expressed in the superficial zone, and less in the middle zone. According to these findings, irisin exhibits differential expression patterns during cartilage development, which reveals that Irisin may be involved in the regulation of cartilage development.

Cartilage tissues were collected from both healthy individuals and OA patients. Histomorphometry of the cartilage structure was observed in the H&E stained slides. Meanwhile, Safranin O staining was performed to identify the changes within the articular cartilage (Figure 1B). Safranin O staining revealed a decrease of cartilage matrix secretion in OA patients. Immunohistochemical results showed that irisin expression decreased in the cartilage of OA patients (Figures 1B,C). In addition, cartilage of OA patients had a decreased irisin mRNA expression compared with healthy humans (Figure 1D). There was also decreased expression of irisin in OA mice compared with sham-operated mice especially in areas of severe cartilage loss (Figures 1E,F). It appears that OA might make a contribution to the loss of cartilage in mice.

### Irisin Inhibits OA Progression in Surgically Induced Mouse Model

**FIGURE 2:**
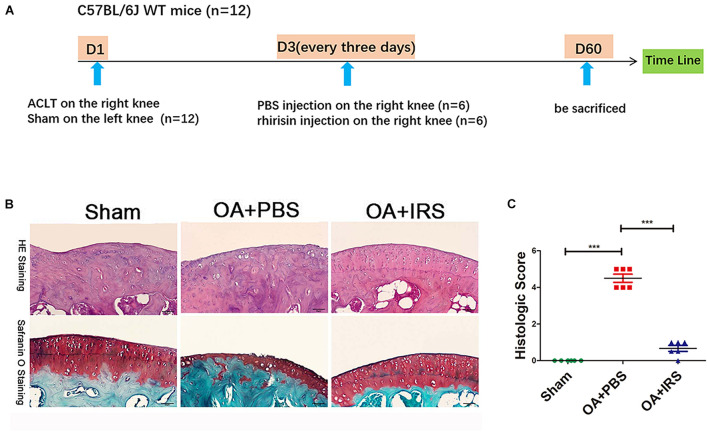
Irisin can significantly inhibit cartilage degradation. **(A)** Timeline of recombinant irisin (r-IRS)/PBS intraarticular injection on anterior cruciate ligament transection (ACLT) mice model. **(B)** Cartilage of OA mice (C57BL/6J wide type mice with ACLT-model, *n* = 6) exhibited significant erosion compared with sham operated mice (C57BL/6J wide type mice, *n* = 6). When intraarticular injected with irisin, mouse cartilage erosion was inhibited (H&E staining and safranin-O staining). **(C)** Histologic score of mouse joints shows OA mice suffer significant erosion, which can be rescued with irisin injection. ***p < 0.001.

The 12-week-old C57BL/6J mice were divided randomly into two groups: ACLT-operated treated with PBS (*n* = 6), and ACLT-operated treated with irisin (*n* = 6) by intraarticular injection (Figure 2A; Pitcher et al., 2016). The ACLT-operated mice treated with PBS group showed a significant decrease in the thickness of cartilage layers compared with the sham-operated group (Figure 2B). The thickness of cartilage of the ACLT-operated mice treated with irisin was similar to the sham-operated group. The OARSI scoring system (Glasson et al., 2010), which is a histologic semi-quantitative of cartilage erosion, showed OA mice exhibited significant erosion in PBS injection group, which was rescued by the irisin injection (Figure 2C). The results indicate that irisin attenuates OA progression in ACLT mice.

### Irisin Inhibits Inflammation and Promotes Anabolism in ADTC5 Cells

**FIGURE 3:**
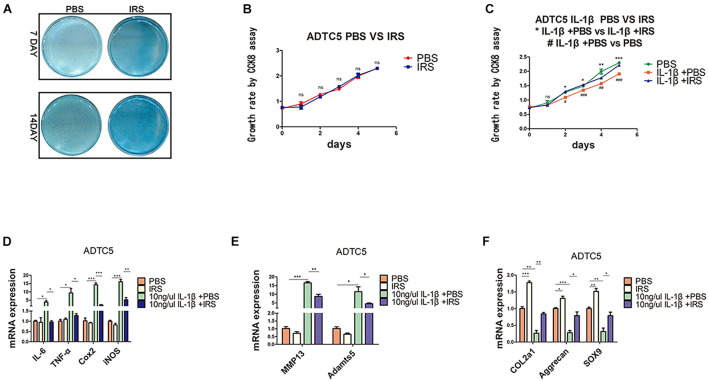
Chondrogenic differentiation, proliferation and inflammation changes in ADTC5 cells. **(A)** Alcian Blue staining of ADTC5. **(B,C)** CCK8 assay reveals the change of cell proliferation *in vitro* (^ns^*p* > 0.05, ^*⁣/⁣*⁣*⁣/⁣*⁣**^ means the comparison between group IL-1β + PBS and group IL-1β + irisin, ^#/##/###^ means the comparison between group IL-1β + PBS and group PBS, *,^#^*p* < 0.05, **,^##^*p* < 0.01, ***,^###^*p* < 0.001). **(D–F)** Gene expression of inflammatory cytokines, inflammatory mediators, catabolic enzymes and anabolic genes expression in ADTC5 cells (^ns^*p* > 0.05, *p < 0.05, **p < 0.01, ***p < 0.001).

ADTC5 cells were preincubated with 100 ng/mL of r-IRS for 24 h before treatment with 10 ng/mL rhIL-1β thereafter for another 8 h. Results revealed that irisin decreases the inflammatory genes expression of IL-1β (interleukin-1 beta), IL-6 (interleu5 cells were preincubated with 100 ng/mL of r-IRS for 24 h before treatment with 10 ng/mL rhIL-1β thereafter for another 8 h. Results revealed that irisin decreases the inflammatory genes expression of IL-1β (interleukin-1 beta), IL-6 (interleukin-6), TNF-α (tumor necrosis factor-alpha), COX2 (catalyzed by cyclooxygenase 2), iNOS (inducible nitric oxide synthase) and catabolic genes expression of MMP13 (matrix metalloproteinase 13) and Adamts5 (Figures 3D,E). Meanwhile, it rescued the proliferation decline caused by rhIL-1β, although irisin had no significant effect on the proliferation of ATDC5 cells (Figures 3B,C). Lastly, we tested the impact of irisin on cell differentiation and anabolism. Cells were incubated for 7and 14 days. Alcian blue staining and qPCR assays showed that irisin significantly promotes anabolism of chondrocytes indicated by the expression of marker genes including Col2a1, Aggrecan and SOX9 (Figures 3A,E).

### Irisin Knock-Out Mice Develop Severe OA While Intra-Articular Injection of Irisin Attenuates DMM-Induced OA Progression

**FIGURE 4:**
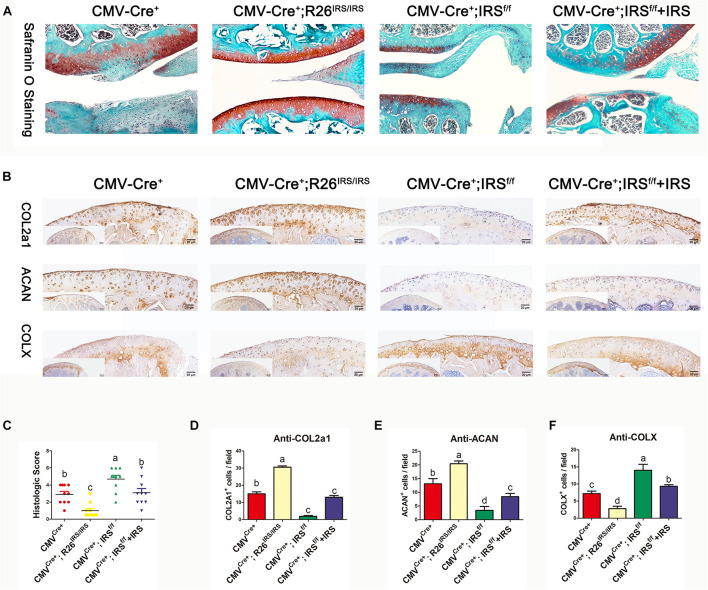
Immunohistochemistry analyses of articular cartilages in medial meniscus (DMM) OA model mice (*n* = 6). **(A)** Safranin O staining. **(B)** IHC staining. **(C)** Osteoarthritis Research Society International (OARSI) scores in mouse DMM models. **(D–F)** Immunostaining positive cells were counted. The comparing data between groups shown in lowercases (a, b, c, and d) are mean values with statistical significance (*P* < 0.05).

**FIGURE 5:**
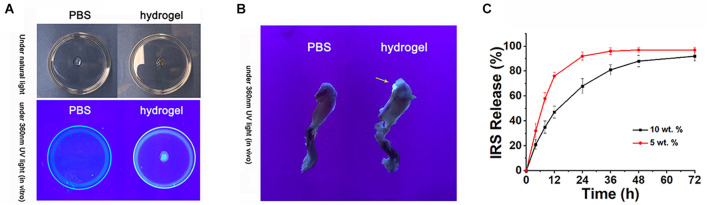
Intraarticular injection of r-IRS protein with a nano-hydrogel. **(A,B)** The injectable citrate-presenting polyester hydrogel can be detected under 360 nm UV light both *in vitro* and *in vivo*. **(C)** rIRS release profile from hydrogel.

CMV-cre mediated IRS KI mice (Supplementary Appendix Figures 1A,B) and KO mice (Zhu et al., 2021) were generated and identified. Irisin was highly expressed in a variety of tissues and organs of CMV^Cre+^; R26^IRS/IRS^ mice, including cartilage, bone, tooth, adipose and muscle compared with control mice (Supplementary Appendix Figure 1C). Similarly, irisin expression was completely knocked out in the same tissues and organs of CMV^Cre+^; IRS^f/f^ mice with no mRNA expression of irisin (Supplementary Figure 1D). When analyzing the OA phenotypes, the IRS KO mice showed more severe OA characteristics than that of age-matched control mice as shown by safranin O staining (Figure 4A), upregulated COLX expression (Figures 4B,F), downregulated COL2a1 (Figures 4B,D) and Aggrecan (Figures 4B,E) expression and OARSI scores (Figure 4C). Then we performed intraarticular injection of r-IRS protein with a nano-hydrogel. The hydrogel possesses ideal biocompatibility, biodegradability, tunable stiffness, intrinsic photoluminescence and injectable properties (Tsou et al., 2018). Besides, the hydrogel has an excellent r-IRS protein release characteristic (Figure 5C) and can be detected under 360 UV light both *in vitro* (Figure 5A) and *in vivo* (Figure 5B). While overexpression IRS both in IRS KI transgenic mice and by intraarticular injection of r-IRS protein with a novel hydrogel (Figure 5), the OA phenotypes were attenuated (Figure 4). These data suggest that IRS plays a protective role in OA progression.

### Irisin Promotes Cartilage Proliferation and Matrix Gene Expression While Inhibiting Inflammation in Primary Mouse Chondrocytes

**FIGURE 6:**
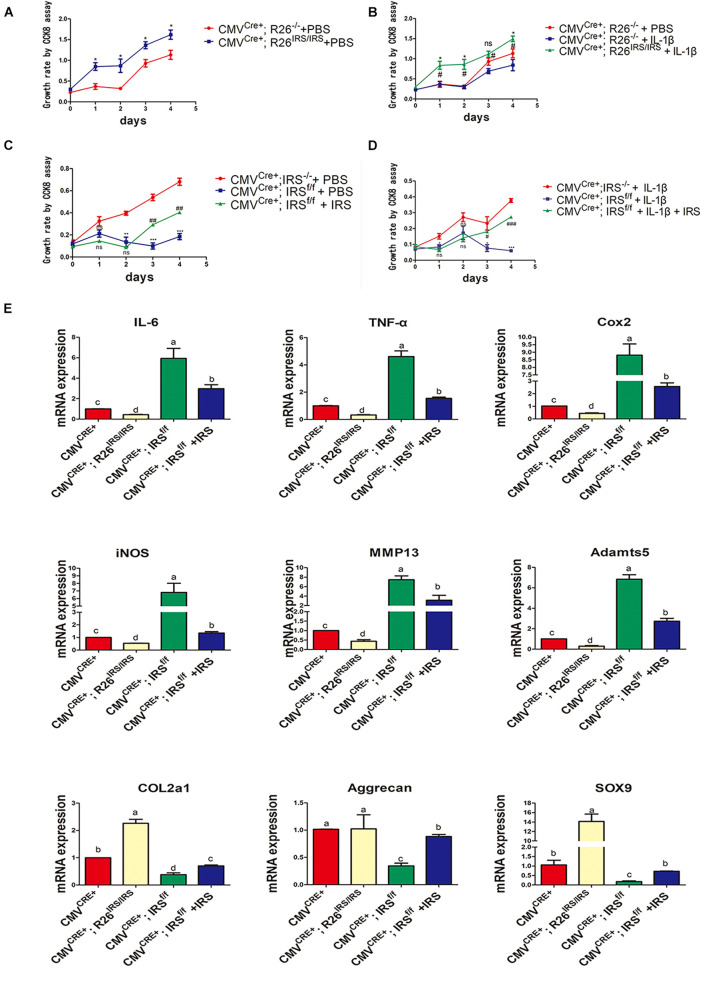
Proliferation and inflammation genes expression of primary chondrocytes isolated from irisin KO and KI mice. **(A)** Proliferation change in irisin KI mice in normal condition (*p < 0.05). **(B)** Proliferation change in irisin KI mice in inflammation conditions induced by IL-1β (^ns^*p* > 0.05, *CMV^Cre+^; R26^− /−^ + IL-1β vs. CMV^Cre+^; R26^IRS/IRS^ + IL-1β, ^#^CMV^Cre+^; R26^− /−^ + PBS vs. CMV^Cre+^; R26^IRS/IRS^ + IL-1β, *,^#^*p* < 0.05). **(C)** Proliferation change in irisin KO mice in normal (^ns^*p* > 0.05, *CMV^Cre+^; IRS^− /−^ + PBS vs. CMV^Cre+^; IRS^f/f^ + PBS, ^#^CMV^Cre+^; IRS^f/f^ + PBS vs. CMV^Cre+^; IRS^f/f^ + PBS + IRS, **,^##^*p* < 0.01, ***,^###^*p* < 0.001). **(D)** Proliferation change in irisin KO mice in inflammation conditions induced by IL-1β (ns *p* > 0.05, *CMV^Cre+^; IRS^− /−^ + IL-1β vs. CMV^Cre+^; IRS^f/f^ + IL-1β, ^#^CMV^*Cre*+^; IRS^f/f^ + IL-1β vs. CMV^Cre+^; IRS^f/f^ + IL-1β + IRS, *,^#^*p* < 0.05, ***,^###^*p* < 0.001). **(E)** Some inflammatory cytokines, inflammatory mediators, catabolic enzymes and anabolic genes expression both in primary chondrocytes isolated from irisin KO and KI mice. The comparing data between groups shown in lowercases (a, b, c, and d) are mean values with statistical significance (*P* < 0.05).

Primary chondrocytes were isolated from gene manipulated and wild type mice. The CCK8 assay showed that proliferation of the cells from irisin KI mice was increased compared with control both in normal and inflammation conditions (Figures 6A,B). Meanwhile, proliferation abilities of the cells from irisin KO mice were decreased compared with the control mice both in normal and inflammatory conditions induced by rhIL-1β, which was rescued by treating with r-IRS (Figures 6C,D). Inflammatory factors (IL-1, IL-6 and TNF-α) and inflammatory mediators (COX2 and iNOS) gene expression level were decreased in irisin KI chondrocytes and increased in irisin KO mice compared with the control under inflammatory condition induced by rhIL-1β (Figure 6E). A set of cartilage anabolic marker genes (COL2a1, Aggrecan and SOX9) expression were increased in irisin KI chondrocytes and decreased in irisin KO mice (Figure 6E).

## Discussion

Traditionally, OA was considered as a “wear-and-tear” disease that led to the loss of articular cartilage as patients age. It is accepted that OA is a multifactorial disorder in origin. Both chronic low-grade inflammatory and biomechanical whole-organ disease processes play an important role in OA pathogenesis. Family history, gender, genetics, diabetes, systemic infection, and innate immunity can also contribute to OA development (Wu et al., 2014). The theory of a “self-perpetuating cycle of joint degeneration” characterizes the pathogenesis of OA. The breakdown of the extracellular matrix (ECM) activates innate immunity and a cyclic cascade of inflammatory events, leading to further joint damage (Zhang et al., 2011b; Luo et al., 2012; Meng et al., 2014; Wu et al., 2014). In general, the cartilage degradation and degeneration, aberrant subchondral bone metabolism, and synovial inflammation contribute together to the pathophysiology of OA. These attributes are also the main targets of multiple OA drugs and therapies. In our study, we investigated the therapeutic impact of irisin and its underlying mechanism. We focused on reducing cartilage degeneration, promoting chondrocyte proliferation and inhibiting inflammation. We first detected the irisin expression pattern during cartilage development, which indicated that irisin is potentially involved in this process. Then, the decreased expression of irisin in OA in both human and mouse indicated that irisin has a negative correlation with OA features. Our irisin intraarticular injection experiment verified our hypothesis that irisin can attenuate pathological features of osteoarthritis and may possess therapeutic potential in treating OA.

To characterize the specific functions of irisin in OA pathogenesis *in vivo*, we generated irisin conditional knockout mice (IRS^f/f^) and irisin over-expressing mice (R26^IRS/IRS^). When mated with CMV^Cre+^ mice, irisin was knocked out or overexpressed systemically, which led to the deletion or overexpression of irisin in the offspring mice. We chose the mice from CMV^cre+^, the CMV^cre+^/IRS^–/–^and the CMV^cre+^/R26^–/–^ lines as the control groups for the KO or KI groups. Deletion of irisin resulted in increased cartilage erosion and collagen degradation, which was in accord with the lessened cartilage damage and collagen degradation in the irisin KI mice.

Two different osteoarthritis surgical models were performed in this study, ACLT and DMM. There is a poorer joint stability in ACLT-model mice compared with the DMM-model mice. Thus, the ACLT-model mice have a faster disease progression in which we can test whether the rh-irisin has an effect in a short period of time. Thus, we used this model in the beginning of the experiments. Compared with the ACLT-model, the DMM-model has a more moderate disease progression which is more like the disease progression of OA patients. Thus, we used the DMM-model mice to test the effect of the rh-irisin in the transgenic mice and the following experiment.

In our study, we used the hydrogel to deliver the rh-irisin. A hydrogel is a 3D network of polymer chains that are hydrophilic, a colloidal gel in which water is the dispersion medium. It is widely used for tissue engineering and drug delivery. In our experiment, by combining irisin-hydrogel and IA injection, we took advantage of the hydrogel with its drug release and biologically active features. With hydrogel-facilitated sustained release, optimal concentration, frequency, and duration, the recombinant irisin was delivered locally to the OA disease site. Injectable hydrogels can be administered via minimally invasive procedures, appropriately fill irregular-shaped defects by acting as 3D scaffolds, and provided a highly hydrated tissue-like environment for cell and tissue growth in our case for promoting cartilage surface repair. However, there are some limitations in this study. We treated the ACLT/DMM model mice with the rh-irisin nearly immediately after injury, which suggested the role of irisin in disease progression rather than a disease treatment.

According to the literature, irisin has distinct effects on cell proliferation of different cell types. For example, irisin can promote the proliferation of C2C12 myoblasts and human periodontal ligament cells (Lee et al., 2019; Pullisaar et al., 2020). Irisin stimulates cell proliferation by targeting the PI3K/AKT pathway in human hepatocellular carcinoma (Shi et al., 2017), while it suppresses cell proliferation of MCF-7 and MDA-MB-231 breast cancer malignant cell lines (Gannon et al., 2015) and pancreatic cancer cells (Liu et al., 2018). In our research, the cell proliferation promoted by irisin in the ADTC5 cells and primary chondrocytes exhibited distinct patterns, indicating that irisin can promote primary chondrocyte proliferation both in normal and inflammatory conditions (Figure 6). However, irisin can only promote ADTC5 cell proliferation under inflammatory conditions (Figures 1B,C). This may partially be due to inhibitory effects of inflammation on the actions of irisin (Figure 1D). Irisin is a multifunctional protein that has beneficial impact on tissue and organ homeostasis (Korta et al., 2019). Irisin has been previously reported to reduce systemic inflammation (Korta et al., 2019), and the expression and release of pro-inflammatory cytokines can be suppressed by irisin in obese individuals (Mazur-Bialy et al., 2017a), where chondrocyte proliferation was promoted, and inflammation inhibited.

The role of irisin in bone formation and regeneration has been examined in previous studies including ours (Zhang et al., 2017; Zhu et al., 2021). Its potential role in chondrocyte metabolism and the various facets of pathogenesis of OA is just emerging. According to the literature, irisin can maintain chondrocyte survival and ECM synthesis by repressing Wnt3a to control autophagic and apoptotic programs. Beyond that, irisin may stimulate proliferation and anabolism inhibiting catabolism of human osteoarthritic chondrocytes (Vadalà et al., 2020). Although the actions and biological functions of irisin in cartilage cells associated with OA were revealed in this study, the potential pathways related to chondrogenesis, proliferation and inflammation during OA pathogenesis are still to be elucidated in details. To clearly delineate the underlying mechanisms, signal pathways such as Wnt, MAPK, AMPK, TGF-β, and NF-KB will be further investigated in our ongoing experiments. It was recently reported that irisin functions through integrin receptors αV/β5 to promote osteocyte survival with relevant sclerostin secretion (Kim et al., 2019). Similar studies for chondrocytes are underway in our laboratory.

## Conclusion

In conclusion, our findings demonstrate that irisin is involved in cartilage development and OA pathogenesis, and that the aberrant alteration of irisin expression in OA cartilage may imply that irisin could be a promising therapeutic target for treating bone and cartilage disorders including OA and rheumatoid arthritis (RA).

## Data Availability Statement

The original contributions presented in the study are included in the article/Supplementary Material, further inquiries can be directed to the corresponding author/s.

## Ethics Statement

Human subjects research was performed according to the Institutional Review Boards at New York University Medical Center (IRB Study Number i11-01488). The animal study was reviewed and approved by Institutional Animal Care and Use Committee (IACUC) of Tufts.

## Author Contributions

XL: conception and design, collection and assembly of data, data analysis and interpretation, and manuscript writing. XZ, HW, and WF: collection and assembly of data, data analysis, and interpretation. TV, XX, and EM: data analysis and interpretation. CL, QT, DH, and JC: conception and design, collection and assembly of data, data analysis and interpretation, administrative support, and manuscript writing. All authors contributed to the critical review of the article for important intellectual content and approved the submitted version.

## Conflict of Interest

The authors declare that the research was conducted in the absence of any commercial or financial relationships that could be construed as a potential conflict of interest.

## Publisher’s Note

All claims expressed in this article are solely those of the authors and do not necessarily represent those of their affiliated organizations, or those of the publisher, the editors and the reviewers. Any product that may be evaluated in this article, or claim that may be made by its manufacturer, is not guaranteed or endorsed by the publisher.
