# Supplementary material for: Roles and Mechanisms of Irisin in Attenuating Pathological Features of Osteoarthritis
Source: Front Cell Dev Biol. 2021 Sep 28;9:703670. doi: 10.3389/fcell.2021.703670 (PMC8509718; doi:10.3389/fcell.2021.703670)
Supplement: Supplementary file 1 [file Table_1.docx]

**Roles and Mechanisms of Irisin in Attenuating Pathological Features of Osteoarthritis**

**Appendix figure**


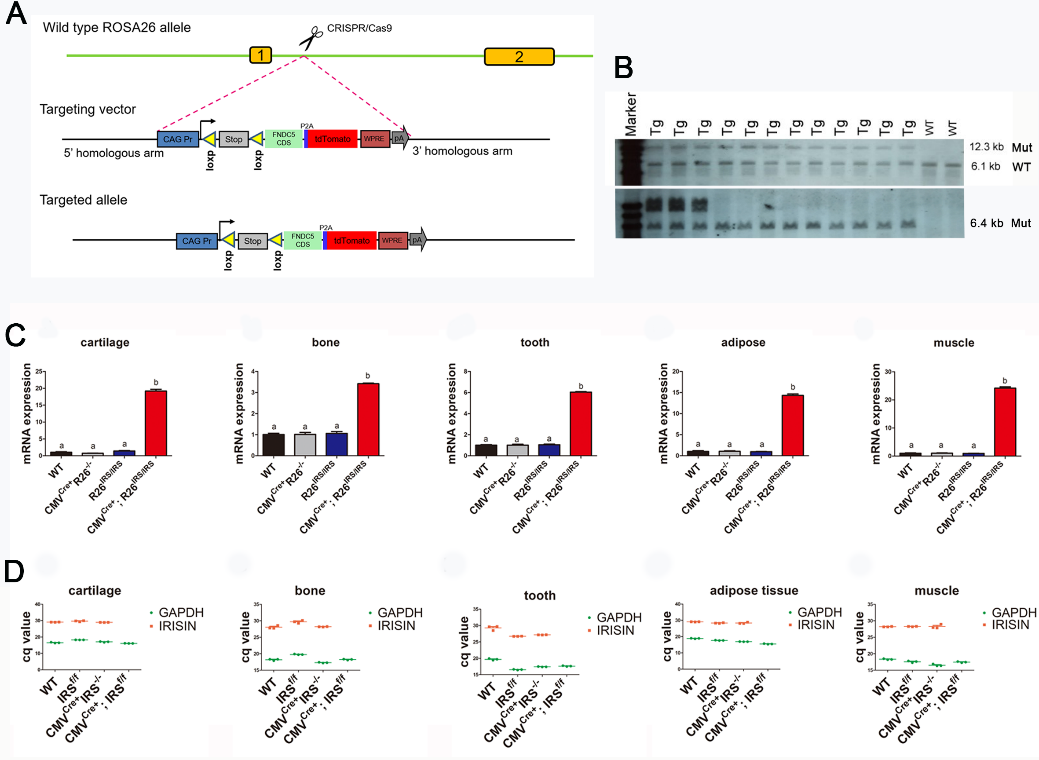


**Appendix Fig. 1**. Establishment of irisin gene manipulated mice: CMV-cre irisin KO mice and KI mice respectively. (A) Schematic representation of the wild type and mutant/targeted alleles in R26^IRS/IRS^ mice. (B) Southern blot was performed to identify the R26^IRS/IRS^ ES cells. (C) Irisin is highly expressed in a variety of tissues and organs, such as cartilage, bone, tooth, adipose, and muscle. (D) Irisin is knocked out in a variety of tissues and organs. The different lowercases (a, b, c and d) mean P<0.05. The same lowercases (a, b, c and d) mean P>0.05.
